# Motivations matter: moral and health-related motives indirectly relate to differential psychological health indicators among vegetarians

**DOI:** 10.1186/s41043-024-00534-2

**Published:** 2024-04-25

**Authors:** Xiaoyu Dai, Angela K.-Y. Leung

**Affiliations:** School of Social Sciences, Singapore Management University, 10 Canning Rise, #04-11, Singapore, 178973 Singapore

**Keywords:** Vegetarianism, Health motivation, Moral motivation, Mental health, Well-being

## Abstract

Due to rising popularity of vegetarianism in recent years, research interest has surged in examining the relationship between vegetarianism and psychological health. However, given inconsistent findings in prior research, the answer to whether practicing vegetarianism is associated with better or worse psychological health is still elusive. The present investigation aimed to demonstrate that vegetarians are not homogeneous in terms of psychological experiences, such that it is crucial to consider the motives behind vegetarians’ dietary choice when examining their psychological health. In a survey study with 266 vegetarians and 104 omnivores, it was shown that health vegetarians displayed higher levels of disordered eating as compared to moral vegetarians and omnivores. Mediation analyses further revealed that, among vegetarians, health motivation was positively correlated with disordered eating tendencies, indirectly linking it with poorer psychological health; moral motivation was positively correlated with prosocial behavior, which in turn predicted better psychological health. These findings have implications for understanding the psychological health of vegetarians with different dietary motives and for developing interventions to promote their psychological health.

## Introduction

Vegetarians constitute a significant portion of the general population in various countries—the rate of vegetarians is estimated to be 28% in India [65], 10% in New Zealand, Australia, and Israel [21], 8% in the U.K. [10], 7% in the U.S. [46], 5% in France [10, 57], 4.3% in Germany (Robert [59]), 3% in Spain [10, 57], and 2.3% in Canada [71]. With the number of vegetarians rising in recent years worldwide, vegetarianism has emerged as an increasingly important social phenomenon that warrants more scientific investigation from a psychological perspective [11, 65]. While the physical health benefits of a properly planned vegetarian diet have been established (see [12], for a review), the relationship between vegetarianism and *psychological* health was relatively less studied, and if studied, the findings were inconsistent. In this light, the present research set out to examine whether and how two key dietary motivations—moral- and health-related motivations—would be associated with differential psychological health among vegetarians.

### Vegetarianism and psychological health: inconclusiveness of existing findings

Existing research has yielded equivocal evidence regarding the association between consuming a vegetarian diet and psychological health. Some studies suggest a connection between vegetarianism and compromised psychological health. For example, a large-scale cross-sectional research of over 9000 young Austrian adult women indicated that vegetarian women had higher rates of depression compared to non-vegetarian women [2]. In a large-scale (*N* > 9000) cross-sectional study of male partners of pregnant women, self-identified vegetarian men exhibited higher depression scores and a greater risk for post-natal depression than non-vegetarian men after adjusting for potential confounding variables [30]. Moreover, in a longitudinal study conducted by Lavallee et al. [45], vegetarianism was associated with slight increases in anxiety and depression over time among a large sample (*N* > 12,000) of Chinese university students.

Conversely, some other research, including cross-sectional and experimental studies, showed a positive association between consuming a vegetarian diet and psychological health. A cross-sectional study [8] of Seventh Day Adventist adult men and women indicated that vegetarians had lower levels of negative emotion than omnivores. Additionally, in a cross-sectional study of vegans, Beezhold et al. [9] found a connection between meat abstention and better psychological health, with vegan men having lower anxiety scores than omnivore men, and vegan women having lower stress scores than omnivore women. Furthermore, in an experimental study [7], omnivore participants experimentally assigned to eat a vegetarian diet (i.e., avoiding meat, fish, and poultry) showed improved mood after two weeks of intervention.

To clarify the relationship between practicing vegetarianism and psychological health, systematic reviews and meta-analyses have therefore been conducted. However, whether vegetarianism is associated with better or worse psychological health is still a matter of debate due to inconclusive nature of the findings. Dobersek et al.’s [17] systematic review of 18 studies indicated higher risks of depression and anxiety in vegetarians relative to omnivores. In contrast, a meta-analysis of 13 studies conducted by Iguacel et al. [38] suggested a positive association between vegetarianism and psychological health, showing that vegetarian diet was associated with lower levels of anxiety and not significantly associated with depression. Notably, heterogeneity (i.e., variations in results across studies) as indexed by I^2^ was high in the meta-analysis, suggesting that vegetarian diets may be associated with differential psychological health among different subgroups of participants.

In another meta-analysis of 13 studies, Ocklenburg and Borawski [54] found that vegetarians had higher depression scores than non-vegetarians, and between-study heterogeneity was high. A meta-analysis of 16 studies conducted by Fazelian et al. [23] also lent support to a positive association between a vegatarian diet and depression, with subgroup analyses revealing several sources of heterogeneity: the type of vegetarian diet, the geographic region where the study was conducted, and people’s age. Conversely, in a recent meta-analysis encompassing 13 studies, Askari et al. [1] found no association between the consumption of a vegetarian diet and depression or anxiety.

In sum, there exist large variations in results across studies on vegetarianism and well-being, and it remains inconclusive whether vegetarianism is associated with poorer or better psychological health. Moreover, it is unclear what might be the psychological mechanism(s) underlying the differential associations between vegetarianism and psychological health, even though some sources of heterogeneity have been revealed (e.g., age, the type of vegetarian diet) [23, 35]. We contend that differences in dietary motives among vegetarians could potentially explain why both a positive and negative association between vegetarianism and psychological health can emerge.

### Primary motivations for vegetarianism: moral and health motivation

Primary motivations for vegetarianism can be classified into two categories [34, 61, 66]: moral motivation (i.e., for animal welfare, for the environment) and health motivation (i.e., for general physical wellness, for weight control). Health vegetarians, that is, individuals who adopt a vegetarian diet for health reasons, are clearly distinct from moral vegetarians, that is, those who adopt a vegetarian diet for moral reasons. In an interview study, Jabs et al. [39] found that the decision of adopting a vegetarian diet among health vegetarians was mainly based on the perceived threat of disease and an analysis of the benefits and barriers to practicing vegetarianism; in contrast, moral vegetarians came to adopt a vegetarian diet when they realized that meat consumption was incongruent with their values of compassion and environmental protection. This finding alludes to key distinctions between health-motivated and morality-motivated vegetarianism. First, health vegetarians may base their dietary choice on a cost–benefit analysis, whereas moral vegetarians may regard vegetarianism as worthwhile in itself. Second, vegetarianism driven by personal health reflects a higher focus on personal benefits, whereas vegetarianism driven by animal welfare and environmental conservation reflects a stronger altruistic and ethical character [66].

Due to their disparate psychological tendencies, health and moral vegetarians are likely to vary in their psychological experiences, which can relate to differential levels of psychological health. However, there is a lack of research comparing the psychological health of these two groups. Therefore, an important objective of this research is to examine whether health and moral vegetarians differ in terms of psychological health. Additionally, another major objective of this research is to identify psychological and behavioral mechanisms that could elucidate the associations between health/moral motivation and psychological health among vegetarians. In subsequent sections, we propose some mediators that may link health motivation to poorer psychological health, and link moral motivation to better psychological health.

#### Health motivation and pathological eating

The idea that vegetarianism is linked to increased disordered eating has been supported by research conducted among both adolescents and young adults [60] and across countries [6, 43]. However, a recent systematic review by McLean et al. [50] presented mixed evidence regarding the relationship between vegetarianism and disordered eating. Among the 33 studies they reviewed, 18 studies indicated a positive association between vegetarianism and disordered eating, whereas 16 studies indicated a null association between vegetarianism and disordered eating. In light of the mixed evidence, the present research aimed to highlight the central role of motivation in the emergence of pathological eating among vegetarians.

##### Orthorexia nervosa

Orthorexia nervosa, characterized by a pathological obsession with eating healthy food [44], has been associated with increased stress and depression [26]. Obsessive symptoms commonly reported by orthorectic individuals include harboring intrusive thoughts related to food and health, feeling anxious about dirt and contamination, and displaying ritualized behaviors during food preparation and food consumption [12]. This mental condition is regarded as clinically impairing when it leads to dietary choices that cause malnutrition, severe weight loss, and/or impairment of daily functioning [20].

A recent systematic review [11] supported a positive correlation between consuming a vegetarian diet and orthorexia. In 11 out of the 14 studies reviewed, vegetarians showed higher levels of orthorexia than omnivores, with effect size ranging from small (*d* = − 0.11) to large (η^2^ = 0.17). However, given that vegetarianism can be driven by different motives, consuming a vegetarian diet may not uniformly reflect a fixation on eating healthy food. It is likely that health (but not moral) motivation is positively associated with orthorexia among vegetarians. Supporting this argument, Barthels et al. [4, 5] found that moral motivations were unrelated to orthorexic tendencies among vegans.

##### Dietary restraint

Vegetarian diets, often perceived as low in calories and fat, are commonly considered as an effective tool for weight control [18, 37]. Given the important role of weight management in physical health [22], the adoption of a vegetarian diet among health-conscious individuals may signal a heightened desire for weight control to promote their physical health. This elevated desire for weight control among health-motivated vegetarians may, in turn, predict increased levels of dietary restraint, which refers to the tendency to exert cognitive effort to restrict food intake for weight control [48].

Dietary restraint has been shown to predict increased symptoms of disordered eating and psychological distress [40]. According to restraint theory [29], the tendency to constantly exert cognitive control over food intake can result in diminished sensitivity to internal cues for hunger and satiety, ultimately leading to disinhibition and overeating when there is momentary lapse of cognitive control [68]. Moreover, restrained eaters may show signs of emotional dysregulation because food becomes a source of anxiety and guilt instead of pleasure to them [68]. In this light, the associations between health motivation, weight control desire, and dietary restraint may imply a negative link between health motivation and psychological health among vegetarians.

##### Inflexible eating

Inflexible eating refers to the tendency to exert *rigid control* over eating, which entails a rigid all-or-nothing mentality in adhering to eating rules, and experiencing “a sense of control when meeting such rules and distress when perceiving failures in meeting such rules” ([19], p. 147). As discussed earlier, health vegetarians tend to evaluate the value of a vegetarian diet based on a cost–benefit analysis. Consequently, they may suffer from psychological conflict when they perceive benefits in not following a vegetarian diet. For example, they may experience conflict between the need to adhere to a highly restrictive diet and the desire to enjoy food, as supported by the moderate positive relationship (*r* = 0.42) between health motivation and meat craving among vegetarians [32]. As such, health vegetarians may need to exert strict control over their impulse to eat meat and other “unhealthy food” to ensure that they eat in a truly healthy manner. Consequently, they might be more prone to inflexible eating. In contrast, moral vegetarians tend to feel disgusted at the thought of meat consumption [63], obviating the need to exert control over their eating behavior. They are, therefore, unlikely to display inflexible eating.

Of note, although both dietary restraint and inflexible eating incorporate cognitive control as a central element, these two tendencies are conceptually distinct. Dietary restraint theorized by Herman and Mack [29] focuses on the tendency to control eating behavior without attending to internal states, leading to non-intuitive eating. On the other hand, inflexible eating conceptualized by Duarte et al. [19] focuses on the rigid all-or-nothing mentality when evaluating the success of effortful control, such that even slight deviations from dietary rules are considered as complete failures.

Together, we posited that health motivation among vegetarians could be linked to poorer psychological health due to its positive association with three disordered eating tendencies:

###### Hypothesis 1a

Health (but not moral) motivation is positively associated with orthorexia, which in turn is negatively associated with psychological health among vegetarians.

###### Hypothesis 1b

Health vegetarians show higher levels of orthorexia than moral vegetarians.

###### Hypothesis 2a

Health (but not moral) motivation is positively associated with dietary restraint, which in turn is negatively associated with psychological health among vegetarians.

###### Hypothesis 2b

Health vegetarians show higher levels of dietary restraint than moral vegetarians.

###### Hypothesis 3a

Health (but not moral) motivation is positively associated with inflexible eating, which in turn is negatively associated with psychological health among vegetarians.

###### Hypothesis 3b

Health vegetarians show higher levels of inflexible eating than moral vegetarians.

#### Health motivation and self-focused attention

Gould [25] posited that health-consciousness is similar to self-consciousness, in that both are accompanied by high levels of inward attention. Aligned with this argument, orthorexic individuals have been found to exhibit impaired capacity for external attention, an indicator of excessive focus on the self [44]. In this light, vegetarians with strong health motivation might be highly attentive towards their own health status, such that they may scrutinize their own body and internal states on a frequent basis, even to an excessive extent. On the other hand, unlike vegetarians driven by high health motivation, vegetarians motivated by high moral concerns may be less likely to ruminate upon aspects of the self, given that their persistent adherence to a restrictive diet is driven by an outward focus towards the welfare of animals and/or the environment [64].

Clinical literature suggests that excessive self-focused attention is an antecedent to numerous psychological disturbances, including intensified bodily sensations, elevated emotional reactivity, impaired cognitive performance, and increased maladaptive coping (e.g., enhanced avoidant coping and reduced effortful coping) [75]. In this light, having an excessive self-focus, arguably linked with health (but not moral) motivation, could have negative implications for psychological health.

##### Hypothesis 4a

Health (but not moral) motivation is positively associated with self-rumination, which in turn is negatively associated with psychological health among vegetarians.

##### Hypothesis 4b

Health vegetarians show higher levels of self-rumination than moral vegetarians.

To summarize, owing to its potential associations with disordered eating behavior and excessive self-focused attention, health-oriented vegetarianism is expected to be associated with poorer psychological health. We contend that this is in contrast with morally motivated vegetarianism, which may be associated with better psychological health due to increased dietary adherence and prosociality. We discuss these associations next.

##### Hypothesis 5a

Health motivation is negatively associated with psychological health among vegetarians.

#### Moral motivation and dietary adherence

Rozin [62] and Rozin et al. [63] posited that morally motivated self-control goals would be associated with more successful regulatory outcomes for two reasons. First, as morally-laden goals are internalized and central to the self, people may accrue more reasons to justify moral actions and thus experience more cognitive consistency when they pursue moral self-control goals. Second, since moral transgressions tend to induce highly aversive emotions such as guilt and disgust, people may be deterred from actions incongruent with their moral values. Following this line of reasoning, vegetarians driven by high moral concerns may show greater dietary adherence because meat abstention to them is a moral ought rather than a personal preference.

High dietary adherence could predict less psychological distress among vegetarians, as they may view low dietary adherence as indicative of self-regulation failure, and hence experience lower self-esteem and more negative emotions [31, 52, 67]. Corroborating this argument, evidence indicated that low dietary adherence can lead to poorer psychological health among consumers of restrictive diets (as reviewed by [15]).

##### Hypothesis 6a

Moral (but not health) motivation is positively associated with dietary adherence, which in turn is positively associated with psychological health among vegetarians.

##### Hypothesis 6b

Moral vegetarians show higher levels of dietary adherence than health vegetarians.

#### Moral motivation and prosociality

Whereas health motivation might be linked with an inward focus on bodily aspects of the self, consuming a vegetarian diet out of moral concerns (for animal welfare and the environment) may reflect genuine care and regard for others. Therefore, vegetarians driven by strong moral motivation may feel more *volitionally* motivated for prosocial acts and engage in prosocial behavior more frequently. Vegans, who tend to be more morally oriented than lacto-ovo vegetarians, reported greater trait empathy than lacto-ovo vegetarians [41]. This finding suggests that more morally oriented vegetarians tend to show higher levels of trait empathy, which was shown to be a powerful predictor of autonomous prosocial motivation and a moderate predictor of actual prosocial behavior [56].

Elevated prosocial tendency may reduce psychological distress and help people experience enhanced well-being. Gilbert [24] argued that prosocial motive which reflects a genuine interest in caring for others (i.e., autonomous prosocial motive) is associated with reduced vulnerability to psychopathology. Besides, prosocial behavior may have incremental values in enhancing well-being above and beyond autonomous prosocial motive. By engaging in prosocial behavior, people can not only exert their personal influence that directly leads to positive changes but also promote closeness to others [49]. Put differently, people can satisfy their needs for competence and relatedness, which are central to people’s well-being according to the self-determination theory [16], by engaging in prosocial acts.

##### Hypothesis 7a

Moral (but not health) motivation is associated with higher levels of autonomous prosocial motivation and prosocial behavior, which in turn are associated with better psychological health among vegetarians.

##### Hypothesis 7b

Moral vegetarians show higher levels of autonomous prosocial motivation and prosocial behavior than health vegetarians.

In a nutshell, considering its positive associations with dietary adherence and prosociality, we expect a positive relationship between moral motivation and psychological health among vegetarians. Moreover, as it is hypothesized that health motivation is negatively associated with psychological health, we expect that moral vegetarians would have better psychological health than health vegetarians.

##### Hypothesis 8a

Moral motivation is positively associated with psychological health among vegetarians.

##### Hypothesis 8b

Moral vegetarians show higher levels of psychological health than health vegetarians.

## Research overview

**Fig. 1:**
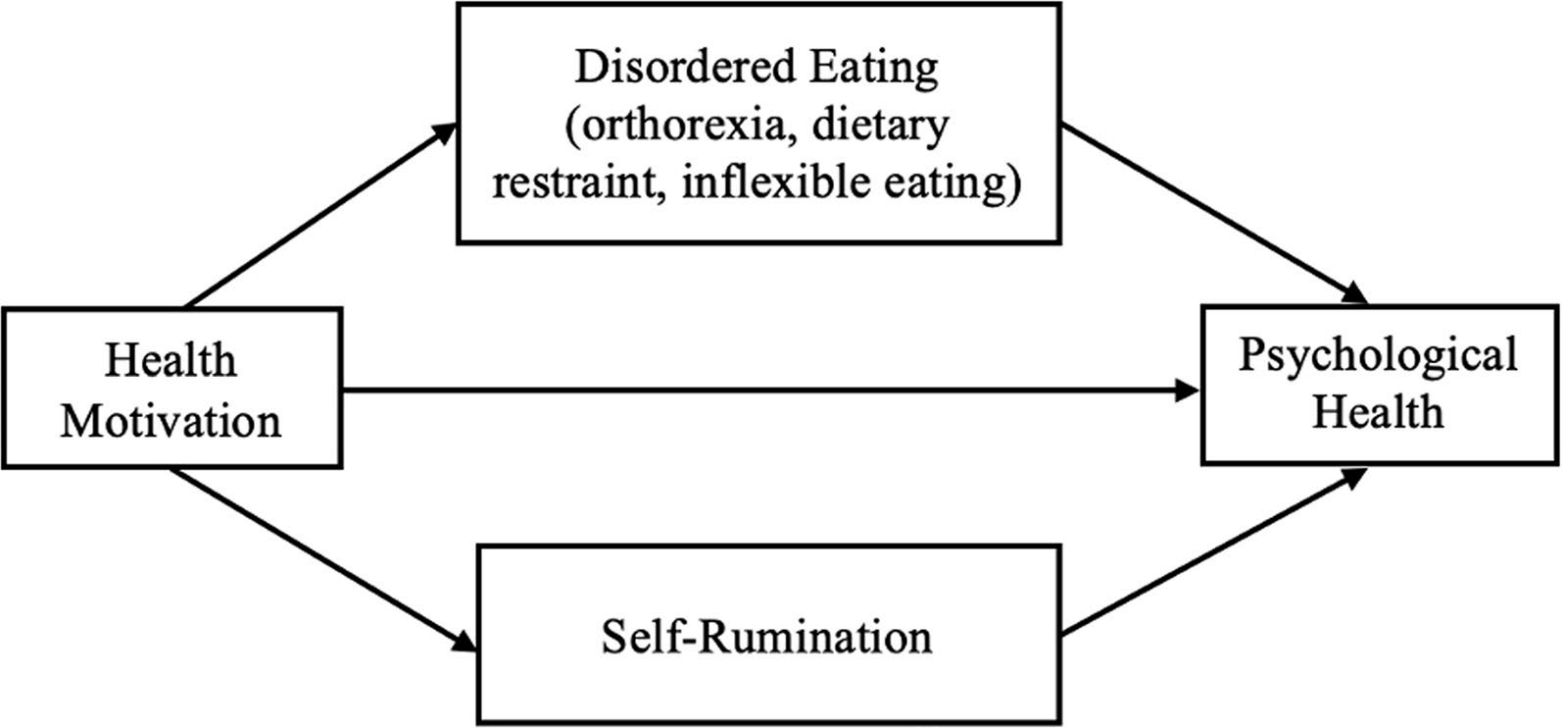
Mediational pathways from health motivation to psychological health

**Fig. 2:**
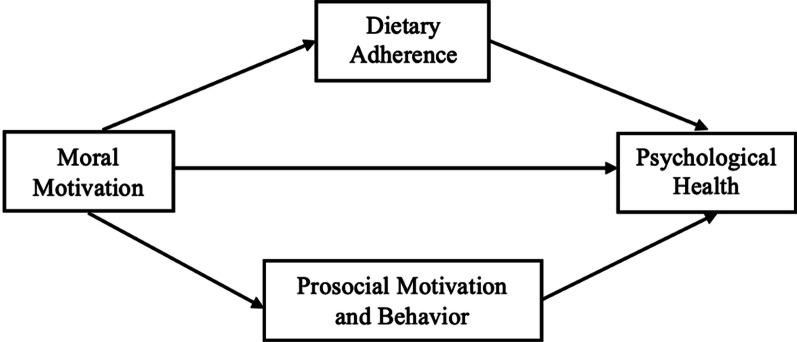
Mediational pathways from moral motivation to psychological health

The present research sought to further differentiate vegetarian individuals based on their primary dietary motivation, such that vegetarians were classified into two groups based on whether they are primarily motivated by moral or health-related concerns. Omnivores were included as a control comparison. ANCOVAs were conducted to compare moral vegetarians, health vegetarians, and omnivores on several psychological and behavioral characteristics, as well as on measures of psychological health while controlling for covariates. In addition, mediation analyses were conducted to examine the hypothesized mediation models, where moral and health motivation as continuous variables are indirectly linked to differential psychological health (see Figs. 1, 2). All data analyses were conducted in IBM SPSS Statistics 29.0.

## Methods

In the current research, health and moral motivation was operationalized in two ways. One way is to assess the moral and health motivation of vegetarians as continuous variables, and this operationalization was used to examine Hypotheses 1a-8a. Health and moral motivation was measured as continuous variables because vegetarianism is often driven by multiple motivations but by different degrees [61]. By examining whether and how health/moral motivation as a continuous variable is related to vegetarians’ psychological health, we acknowledge that both health and moral concerns can motivate vegetarians’ dietary choice instead of conceiving that vegetarians are either motivated by health or moral reasons only.

The second way is to classify vegetarians into moral vegetarians and health vegetarians based on their self-reported primary motivation for vegetarianism, and this operationalization was used to examine Hypotheses 1b, 2b, 3b, 4b, 6b, 7b, 8b. As one important aim of the current research is to address inconclusive findings on the link between vegetarianism and psychological health, health/moral motivation was also measured as a categorical variable so as to enable group comparisons between health vegetarians, moral vegetarians, and omnivores.

### Participants and procedure

Past research [51] comparing vegetarians and omnivores in anxiety and depression indicates a small-to-medium effect size. Assuming a small-to-medium effect size for ANOVA (f = 0.19, α = .05, 1 − β = .80), a priori power analysis by G*Power estimated a sample size of 270 (ninety people for each group). To buffer for low quality data and unbalanced ratio of health vegetarians to moral vegetarians, we planned to recruit 135 omnivores and 270 vegetarians. Sensitivity power analysis by G*Power suggested that with a sample size of 270, a minimum effect size of f^2^ = .03 is required to detect a significant increase in R^2^ for linear multiple regression with 1 tested predictor and 11 total predictors.

Participants received informed consent forms, with all procedures approved by the ethics board of Singapore Management University (IRB-22-062-E034-M1(622)). A total of 411 U.S. residents (141 omnivores and 270 vegetarians) were recruited via MTurk Toolkit managed by CloudResearch, to complete an online survey hosted by Qualtrics. Two screening questions were administered to identify people who (1) self-identity as vegetarians and also (2) consume a vegetarian diet. After the screening questions, vegetarians indicated their primary motivation for following a vegetarian diet. Next, both vegetarians and omnivores indicated the extent to which their dietary choice is driven by health and moral concerns. Then they completed measures of the mediating variables and psychological health before responding to some demographic items.

One Captcha verification question was included at the beginning of the survey to prevent bot responses. Forty-one responses were excluded because of failing the quality check, honesty check, or attention check (“This is an attention check. Choose “4-Somewhat agree” for this item”). We were left with 370 valid responses, with 61.08% participants being female, 77.57% participants being White. The valid responses were from 266 vegetarians (137 moral vegetarians, 93 health vegetarians, 36 other vegetarians) and 104 omnivores. Descriptive statistics of demographic variables and physical health of moral vegetarians, health vegetarians, and omnivores were summarized in Table 1.

**Table 1:** Descriptive statistics of demographic variables and physical health

Dietary group	Moral vegetarians *n* = 137		Health vegetarians *n* = 93		Omnivores *n* = 104	
	*n*	%	*n*	%	*n*	%
*Gender*						
Female	98	71.5	50	53.8	49	47.1
Male	36	26.3	40	43	54	51.9
Non-binary	1	0.7	1	1.1	0	0
Prefer not to say	2	1.5	2	2.2	1	1.0
*Age*						
18–24	13	9.5	3	3.2	6	5.8
25–34	51	37.2	26	28.0	38	36.5
35–44	40	29.2	23	24.7	40	38.5
45–54	17	12.4	17	18.3	9	8.7
55–64	14	10.2	11	11.8	6	5.8
≥ 65	2	1.5	13	14.0	5	4.8
*Ethnicity*						
White	119	86.9	66	71.0	79	76.0
Black	5	3.6	14	15.1	11	10.6
Hispanic	9	6.6	5	5.4	4	3.8
American Indian or Alaska Native	0	0	0	0	1	1.0
Asian	1	0.7	7	7.5	7	6.7
Native Hawaiian or Pacific Islander	0	0	0	0	0	0
Other	3	2.2	1	1.1	2	1.9
*Urbanicity*						
≤ 5000	14	10.2	11	11.8	9	6.8
5000–20,000	21	15.3	13	15.3	24	18.2
20,000–100,000	26	19.0	23	27.1	39	29.5
100,000–500,000	25	18.2	21	24.7	31	23.5
500,000–1,000,000	17	12.4	6	7.1	10	7.6
≥ 1,000,000	24	17.5	11	12.9	19	14.4
I don’t know	10	7.3	9	9.7	6	5.8
*Household income*						
≤ $25,000	24	17.5	17	18.3	11	10.6
$25,000–$49,999	33	24.1	19	20.4	36	34.6
$50,000–$74,999	35	25.5	27	29.0	22	21.2
$75,000–$99,999	15	10.9	12	12.9	14	13.5
$100,000–$149,999	14	10.2	6	6.5	13	12.5
≥ $150,000	13	9.5	9	9.7	6	5.8
Prefer not to say	3	2.2	3	3.2	2	1.9
Physical health	3.47 (0.99)		3.57 (0.81)		3.42 (0.92)	

Figures indicate *n* and % except for physical health where the numbers indicate mean (standard deviation)

### Materials

#### Dietary pattern

Dietary pattern was measured by a single item adapted from Mullee et al. [53]: “Please indicate which of the following best describes your dietary pattern?” Below this question was a list of six responses: (1) Eating meat or fish almost every day or not intentionally abstaining from meat or fish. (2) Intentionally limiting meat or fish to a few times a week. (3) Eating meat or fish no more than once a week. (4) No meat but eating fish. (5) No meat or fish. (6) No animal products. Following past practice [13, 53], participants were considered as omnivores if they chose Option (1), part-time vegetarians if they choose Option (2), semi-vegetarians if they chose Option (3), pesco-vegetarians if they chose Option (4), vegetarians if they choose Option (5), and vegans if they chose Option (6). In the present research, semi-vegetarians, pesco-vegetarians, vegetarians, and vegans were all considered as vegetarians [23], but part-time vegetarians were considered as non-vegetarians because individuals who consume meat more than once a week are considered as non-vegetarians in some literature [13, 33]. Participants who chose Option (2) did not proceed to complete the survey because they were not considered as omnivores or vegetarians. As vegetarians following different diet types were found to differ in terms of psychological health [23], diet type (non-strict vegetarian = 0 vs. strict vegetarian = 1) was controlled for when conducting mediation analyses. Semi-vegetarians and pesco-vegetarians were considered as consuming a non-strict vegetarian diet because they do not completely abstain from eating meat or fish; vegetarians and vegans were considered as consuming a strict vegetarian diet.

#### Self-identification

Participants were asked to indicate whether they consider themselves as a vegetarian/vegan (yes or no). Participants who reported a vegetarian dietary pattern (i.e., choosing Options 3–6 above) but did not self-identify as vegetarian/vegan did not proceed with the survey.

#### Primary motivation for following a vegetarian diet

The most important motivation for consuming a vegetarian diet was measured by one item developed by Hoffman et al. [34]: “The main reason I am (still) a vegetarian is because of ….” Ten options were given: (1) “animal rights,” (2) “physical health,” (3) “the environment,” (4) “religion/spiritual beliefs,” (5) “weight control/loss,” (6) “taste,” (7) “family/friends,” (8) “born and/or raised as vegetarians,” (9) “saving money,” and (10) “other” with a mandatory textbox. As the items asked the *predominant* reason for choosing to become a vegetarian, participants were allowed to choose only one option for each item. Respondents were classified as moral vegetarians if they chose “animal rights” or “the environment” and as health vegetarians if they chose “physical health” or “weight loss/control”. Participants who did not fall into either the category of moral or health vegetarian were removed from statistical comparisons of health vegetarians, moral vegetarians, and omnivores. However, these participants were still included for correlation and mediation analyses.

#### Dietary motive

Participants indicated the extent to which their food choices are driven by health-related motives (i.e., health, weight control; α = .66) and morality-related motives (i.e., environmental protection, animal welfare; α = .84) by responding to four items of the shortened Food Choice Questionaire [55] on a 7-point scale (1 = *not at all important*, 7 = *very important*). A sample item is “It is important to me that the food I eat on a typical day is healthy”.

#### Disordered eating

Three indicators of disordered eating were measured, including (a) orthorexia, (b) dietary restraint, and (c) inflexible eating. To assess orthorexia, participants were asked to rate the ten-item Düsseldorfer Orthorexie Skala (DOS; [3], α = .88) on a four-point scale (1 = *this does not apply to me* to 4 = *this applies to me*). A sample item is “I feel upset after eating unhealthy foods.” Dietary restraint was measured by the 10-item Restrained Eating subscale (e.g., “If you have put on weight, do you eat less than you usually do?”) of the Dutch Eating Behavior Questionnaire (DEBQ; [72], α = .94). The items were rated on a 5-point scale (1 = *never* to 5 = *very often*). Inflexible eating was measured by the Inflexible Eating Questionnaire (IEQ; [19], α = .94). Participants rated 11 items (e.g., “When I do not follow one of my eating rules, then I make an effort to compensate it by following my rules even more strictly”) on a 5-point scale (1 = *fully disagree* to 5 = *fully agree*).

#### Self-rumination

Self-rumination was measured by the 12-item self-rumination subscale of the Rumination-Reflection Questionnaire (RRQ; [70], α = .95). A sample item reads “My attention is often focused on aspects of myself I wish I’d stop thinking about” (1 = *strongly disagree* to 5 = *strongly agree*).

#### Subjective dietary adherence

Subjective dietary adherence was measured by one item: “How successful have you been in consistently eating your chosen diet during the past few weeks?” (1 = *not at all* to 7 = *very successful*), adapted from Perceived Past Healthy Eating Behavior Scale [14].

#### Prosociality

Both autonomous prosocial motivation and prosocial behavioral engagement was measured. Autonomous motivation for prosocial acts was measured by the 4-item Autonomous Prosocial Motivation subscale of the Motivation to Help Scale [73]. Participants were asked to respond to one question: “Why do you do things that help other people?” by rating four items on a 7-point scale (1 = *not at all* to 7 = *very much so*; α = .90). A sample item reads “Because it is an important choice I really want to make”. Prosocial behavior was measured by six items developed by Pavey et al. [56] (α = .84). Participants reported the extent to which they engaged in a range of prosocial activities (e.g., offer money to charities) during the last month on a 5-point scale (1 = *never* to 5 = *very often*).

#### Psychological health measures

Measures of negative and positive psychological health were included. The Depression, Anxiety, and Stress Scale (DASS-21; [47]) was used to measure depression (seven items; e.g., “I felt down-hearted and blue”; α = .95), anxiety (seven items; e.g., “I felt scared without any good reason”, α = .90), and stress (seven items; “I found it hard to wind down”; α = .90) on a 4-point scale (0 = *did not apply to me at all* to 3 = *applied to me very much or most of the time*). To measure positive mental health, the 14-item Mental Health Continuum Short Form (MHC-SF; [42]) was administered. Participants rated the frequency of experiencing various feelings in the past month, including feelings of emotional well-being (three items; e.g., “How often in the past month did you feel happy?”; α = .90), social well-being (five items; e.g., “How often in the past month did you feel that you had something important to contribute to society”; α = .88), and psychological well-being (six items; e.g., “How often in the past month did you feel that you liked most parts of your personality”; α = .89) on a scale from 1 (*never*) to 6 (*every day*).

#### Physical health

Participants rated their physical health on a one-item 5-point scale (1 = *excellent* to 5 = *poor*). Physical health was reversely coded so that higher scores reflect better physical health.

#### Demographics

Participants were asked to indicate their gender, age, ethnicity, household income, and urbanicity (i.e., population size of their resident area). Ethnicity and gender were dummy coded, with minority races and males coded as reference categories.

## Results

### Demographic comparison

Descriptive statistics of demographic variables were computed for the three groups (i.e., health vegetarians, moral vegetarians, and omnivores) and summarized in Table 1.

The Kruskal–Wallis test revealed a significant difference in age among the three groups, χ^2^(2) = 14.09, *p* < .001. Multiple pair-wise nonparametric comparisons with a Bonferroni-type adjustment showed that health vegetarians were significantly older than moral vegetarians and omnivores; moral vegetarians and omnivores did not significantly differ in age. The Kruskal–Wallis test indicated no significant group difference in household income, urbanicity, and physical health.

A chi-square test of independence showed that the proportion of females significantly differed between groups, χ^2^(2) = 17.03, *p* < .001. Post-hoc analysis with Bonferroni correction revealed that the proportion of females was significantly higher in moral vegetarians (73.1%) than in health vegetarians (55.6%) and omnivores (47.6%); the proportion of females did not significantly differ between health vegetarians and omnivores.

### Bivariate correlations in vegetarians

Bivariate correlation analyses were performed for health food choice motive (FCM), moral FCM, and other focal variables (including mediators and measures of psychological health) in vegetarians. The results were summarized in Table 2.

**Table 2:** Correlations between focal variables

Variable	*M*	SD	1	2	3	4	5	6	7	8	9	10	11	12	13	14	15
1. Health FCM	5.53	1.25	–														
2. Moral FCM	5.75	1.28	.26***	–													
3. Orthorexia	2.53	0.63	.50***	.27***	–												
4. Dietary restraint	2.97	0.95	.46***	.07	.36***	–											
5. Inflexible eating	3.09	0.99	.44***	.15*	.68***	.63***	–										
6. Self-rumination	3.35	1.06	− .13*	− .02	− .02	.23***	.18**	–									
7. Dietary adherence	6.14	1.16	.05	.27***	.08	− .10	− .07	− .11	–								
8. Prosocial motivation	5.73	1.18	.19**	.43***	.17**	.18**	.16**	.05	.25***	–							
9. Prosocial behavior	2.64	0.87	.18**	.23***	.14*	.23***	.15*	− .11	− .06	.24***	–						
10. Depression	1.83	0.84	− .05	.002	.09	.18**	.19**	.53***	− .13*	.01	− .04	–					
11. Anxiety	1.61	0.70	− .04	.04	.09	.21***	.20**	.45**	− .20***	.05	.14*	.73***	–				
12. Stress	1.91	0.74	− .05	− .02	.09	.23***	.23***	.59***	− .16**	.06	.04	.78***	.80***	–			
13. Emotional well-being	4.30	1.14	.13*	.07	− .03	− .08	− .13*	− .47***	.16*	.07	.21***	− .68***	− .41***	− .47***	–		
14. Social well-being	3.25	1.31	.15*	.06	.08	− .06	− .01	− .52***	.06	.09	.37***	− .47***	− .25***	− .40***	.68***	–	
15. Psychological well-being	4.13	1.12	.19**	.09	.04	− .08	− .07	− .52***	.14*	.15*	.26***	− .66***	− .43***	− .53***	.80***	.71***	–

*p < .05; **p < .01; ***p < .001

### Mediation analyses in vegetarians

SPSS PROCESS [28] Model 4 was used to test the proposed mediation models (see Figs. 1, 2). Bootstrapping was performed with 5000 samples to test the significance of indirect effects. Mediation analyses were conducted including all vegetarians.

Given that health FCM and moral FCM were significantly correlated (*r*(264) = .26,* p* < .001), moral FCM was statistically controlled for when examining correlations with health FCM and vice versa. Age, gender (male = 0, female = 1), ethnicity (minority race = 0), urbanicity, household income, physical health, and diet type (0 = non-strict vegetarian) were also entered as covariates.

The total effects of health FCM and moral FCM on measures of psychological health were summarized in Table 3. Contrary to Hypothesis 5a, Health FCM did not have a significant total effect on any measure of psychological health. Contrary to Hypothesis 8a, moral FCM did not have a significant total effect on any measure of psychological health.

**Table 3:** Linear regression analysis predicting psychological health—total effects model

Predictor	Outcome	Depression	Anxiety	Stress	EWB	SWB	PWB
		*b*	*b*	*b*	*b*	*b*	*b*
Constant		3.05*** (.38)	2.16*** (.30)	3.29*** (.33)	1.91*** (.49)	1.41* (.57)	1.42** (.48)
Age		− .13** (.04)	− .16*** (.03)	− .15*** (.03)	.08 (.05)	.02 (.06)	.15** (.05)
Gender (0 = male)		.12 (.12)	.13 (.09)	.14 (.10)	.20 (.15)	− .23 (.17)	− .03 (.14)
Ethnicity (0 = non-White)		.18 (.14)	− .06 (.11)	.07 (.12)	− .16 (.18)	− .32 (.21)	− .06 (.18)
Urbanicity		− .002 (.04)	− .004 (.03)	− .02 (.03)	− .003 (.05)	− .01 (.05)	− .02 (.04)
Income		− .09* (.04)	− .08** (.03)	− .07* (.03)	.12* (.05)	.07 (.06)	.06 (.05)
Diet type (0 = non-strict vegetarian		.05 (.13)	− .16 (.10)	− .10 (.11)	− .03 (.16)	− .14 (.19)	− .21 (.16)
Physical health		− .23*** (.07)	− .08 (.05)	− .17** (.06)	.46*** (.08)	.48*** (.10)	.46*** (.08)
Health FCM		.07 (.05)	.03 (.04)	.04 (.04)	− .03 (.06)	.01 (.07)	.03 (.06)
Moral FCM		− .04 (.05)	.01 (.04)	− .03 (.04)	.05 (.07)	.06 (.08)	.07 (.06)

Linear regression predicting measures of psychological health in vegetarians by health and moral FCMs while controlling for covariates (age, gender, ethnicity, urbanicity, household income, diet type, and physical health)

*EWB* Emotional well-being, *SWB* Social well-being, *PWB* Psychological well-being, *FCM* Food choice motive

*p < .05; **p < .01; ***p < .001

#### Disordered eating measures as mediators

To recall, three disordered eating indicators were measured: orthorexia, dietary restraint, and inflexible eating. They were entered as parallel mediators. The results were summarized in Table 4.

**Table 4:** Mediation analysis testing the indirect effects of health motivation on psychological health indicators via disordered eating variables

Predictor	Outcome	Orthorexia	Dietary Restraint	Inflexible Eating	Depression	Anxiety	Stress	EWB	SWB	PWB
		b	b	b	b	b	b	b	b	b
Constant		1.01*** (.25)	1.28** (.40)	1.26** (.41)	2.61*** (.39)	2.16*** (.30)	2.83*** (.32)	2.42*** (.50)	1.80** (.59)	1.91*** (.49)
Health FCM		.24*** (.03)	.39*** (.05)	.36*** (.05)	− .06 (.06)	− .09* (.04)	− .09 (.05)	.12 (.07)	.13 (.08)	.17* (.07)
Orthorexia					.08 (.12)	.07 (.10)	.06 (.10)	− .03 (.16)	.05 (.19)	− .04 .16
Dietary restraint					.15* (.07)	.18** (.06)	.19** (.06)	− .13 (.10)	− .17 (.11)	− .15 (.09)
Inflexible eating					.13 (.09)	.09 (.07)	.12 (.07)	− .25* (.11)	− .17 (.13)	− .20 (.11)
Moral FCM		.03 (.03)	− .06 (.05)	.02 (.05)	− .03 (.05)	.02 (.04)	− .02 (.04)	.05 (.06)	.05 (.08)	.07 (.06)
Age		.01 (.03)	− .07 (.04)	− .04 (.04)	− .12** (.04)	− .14*** (.03)	− .14*** (.03)	.06 (.05)	.002 (.06)	.13** (.05)
Gender (0 = male)		− .16* (.08)	.27* (.12)	− .19 (.12)	.11 (.12)	.11 (.09)	.12 (.10)	.18 (.15)	− .21 (.18)	− .03 (.15)
Ethnicity (0 = non-White)		.11 (.09)	.13 (.15)	.14 (.15)	.14 (.13)	− .10 (.11)	.02 (.11)	− .11 (.17)	− .28 (.21)	− .01 (.17)
Diet type (0 = non-strict vegetarian)		.01 (.08)	− .09 (.13)	− .07 (.13)	.07 (.12)	− .14 (.09)	− .07 (.10)	− .06 (.16)	− .17 (.19)	.47 (.08)
Physical health		.07 (.04)	− .02 (.07)	.03 (.07)	− .23*** (.06)	− .08 (.05)	− .17** (.05)	.46*** (.08)	.48*** (.10)	0.48*** (.08)
Indirect effect via orthorexia					.02 (.04) [− .06, .09]	.02 (.03) [− .04, .07]	.01 (.03) [− .05, .07]	− .01 (.04) [− .08, .07]	.01 (.05) [− .08, .11]	− .01 (.04) [− .09, .07]
Indirect effect via dietary restraint					.06 (.03) [− .002, 0.12]	.07 (.03) [.02, .12]	.07 (.03) [.02, .14]	− .05 (.04) [− .13, .04]	− .07 (.05) [− .17, .03]	− .06 (.04) [− .13, .02]
Indirect effect via inflexible eating					.05 (.04) [− .02, .12]	.03 (.03) [− .02, .08]	.04 (.03) [− .01, .10]	− .09 (.04) [− .18, − .004]	− .06 (.05) [− .16, .05]	− .07 (.04) [− .16, .01]

*EWB* Emotional well-being, *SWB* Social well-being, *PWB* Psychological well-being, *FCM* Food choice motive

*p < .05; **p < .01; ***p < .001

Health FCM did not have significant indirect effects via orthorexia on depression (*b*_indirect_ = .02, *SE*_boot_ = .05, 95% CI_boot_ [− .06, .09]), anxiety (*b*_indirect_ = .02, *SE*_boot_ = .03, 95% CI_boot_ [− .04, .07]), and stress (*b*_indirect_ = .01, *SE*_boot_ = .03, 95% CI_boot_ [− .05, .07]). Its indirect effects via inflexible eating on depression (*b*_indirect_ = .05, *SE*_boot_ = .04, 95% CI_boot_ [− .02, .12]), anxiety (*b*_indirect_ = .03, *SE*_boot_ = .03, 95% CI_boot_ [− .02, .08]), and stress (*b*_indirect_ = .04, *SE*_boot_ = .03, 95% CI_boot_ [− .01, .10]) also did not reach significance. On the other hand, health FCM had significant positive indirect effects via dietary restraint on anxiety (*b*_indirect_ = .07, *SE*_boot_ = .03, 95% CI_boot_ [.02, .12]) and stress (*b*_indirect_ = .07, *SE*_boot_ = .03, 95% CI_boot_ [.02, .14]), but its indirect effect on depression was not significant (*b*_indirect_ = .06, *SE*_boot_ = .03, 95% CI_boot_ [− .002, .12]).

Results showed that the indirect effects of health FCM on measures of emotional well-being via orthorexia (*b*_indirect_ = − .01, *SE*_boot_ = .04, 95% CI_boot_ [− .08, .07]) and dietary restraint (*b*_indirect_ = − .05, *SE*_boot_ = .04, 95% CI_boot_ [− .13, .04]) were not significant. In contrast, health FCM was found to have significant, negative indirect effects on emotional well-being via inflexible eating (*b*_indirect_ = − .09, *SE*_boot_ = .04, 95% CI_boot_ [− .18, − .004]).

The indirect effects of health FCM on social well-being were not significant via orthorexia (*b*_indirect_ = .01, *SE*_boot_ = .05, 95% CI_boot_ [− .08, .11), via dietary restraint (*b*_indirect_ = − .07, *SE*_boot_ = .05, 95% CI_boot_ [− .17, .03]), and via inflexible eating (*b*_indirect_ = − .06, *SE*_boot_ = .05, 95% CI_boot_ [− .16, .05]). The indirect effects of health FCM on psychological well-being via orthorexia (*b*_indirect_ = − .01, *SE*_boot_ = .04, 95% CI_boot_ [− .09, .07]), via dietary restraint (*b*_indirect_ = − .06, *SE*_boot_ = .04, 95% CI_boot_ [− .13, .02]), and via inflexible eating (*b*_indirect_ = − .07, *SE*_boot_ = .04, 95% CI_boot_ [− .16, .01]) were not significant, either.

Inconsistent with Hypothesis 1a, orthorexia did not significantly mediate the relationship between health motivation and any indicator of psychological health. Consistent with Hypothesis 2a, the results suggested that health motivation was positively associated with dietary restraint, which in turn was positively associated with anxiety and stress, which are indicators of negative psychological health. Consistent with Hypothesis 3a, health motivation was positively correlated with inflexible eating, which in turn was negatively correlated with emotional well-being, an indicator of positive psychological health.

We reran the same mediation analyses excluding vegetarians whose primary motivation is neither health nor morality. The results were consistent, except that health FCM was found to have a significant indirect effect on depression via dietary restraint, *b*_indirect_ = .07, *SE*_boot_ = .03, 95% CI_boot_ [.001, .13], after excluding vegetarians whose primary motivation is neither health nor morality.

#### Self-rumination as a mediator

The results were summarized in Table 5.

**Table 5:** Mediation analysis testing the indirect effects of health motivation on psychological health indicators via self-rumination

Predictor	Outcome	Self-Rumination	Depression	Anxiety	Stress	EWB	SWB	PWB
		*b*	*b*	*b*	*b*	*b*	*b*	*b*
Constant		4.84*** (.47)	1.16** (.41)	1.32*** (.34)	1.45*** (.34)	4.21*** (.54)	4.53*** (.60)	3.76*** (.52)
Health FCM		.02 (.06)	.06 (.04)	.03 (.03)	.03 (.03)	− .02 (.05)	.03 (.06)	.04 (.05)
Self-rumination			.39*** (.05)	.26*** (.04)	.38*** (.04)	− .48*** (.06)	− .64*** (.07)	− .49*** (.06)
Moral FCM		− .03 (.06)	− .03 (.04)	.02 (.04)	− .02 (.04)	.04 (.06)	.04 (.07)	.06 (.06)
Age		− .17*** (.05)	− .07 (.04)	− .11*** (.03)	− .09** (.03)	− .01 (.05)	− .09 (.05)	.07 (.05)
Gender (0 = male)		.40** (.14)	− .04 (.10)	.03 (.09)	− .01 (.09)	.39** (.14)	.03 (.15)	.16 (.13)
Ethnicity (0 = non-White)		.08 (.17)	.16 (.12)	− .08 (.10)	.04 (.10)	− .13 (.16)	− .27 (.18)	− .02 (.16)
Urbanicity		− .02 (.04)	.01 (.03)	.01 (.03)	− .02 (.03)	− .01 (.04)	− .03 (.05)	− .03 (.04)
Income		− .04 (.05)	− .08* (.03)	− − .07** (.03)	− .05* (.03)	.10* (.04)	.05 (.05)	.04 (.04)
Diet type (0 = non-strict vegetarian)		.12 (.15)	− .0003 (.11)	− .19* (.09)	− .14 (.09)	.03 (.14)	− .06 (.16)	− .15 (.14)
Physical health		− .28*** (.08)	− .12* (.06)	− .01 (.05)	− .06 (.05)	.32*** (.08)	.31*** (.09)	.33*** (.08)
Indirect effect via self-rumination			.01 (.02) [− .03, .05]	.005 (.01) [− .02, .03]	.01 (.02) [− .03, .04]	− .01 (.02) [− .06, .04]	− .01 (.03) [− .07, .05]	− .01 (.02) [− .06, .04]

*EWB* Emotional well-being, *SWB* Social well-being, *PWB* Psychological well-being, *FCM* Food choice motive

*p < .05; **p < .01; ***p < .001

Health FCM did not have a significant indirect effect on any measures of psychological health via self-rumination: depression (*b*_indirect_ = .01, *SE*_boot_ = .02, 95% CI_boot_ [− .03, .05]), anxiety (*b*_indirect_ = .005, *SE*_boot_ = .01, 95% CI_boot_ [− .02, .03], and stress (*b*_indirect_ = .01, *SE*_boot_ = .02, 95% CI_boot_ [− .03, .04]), emotional well-being (*b*_indirect_ = − .01, *SE*_boot_ = .02, 95% CI_boot_ [− .06, .04]), social well-being (*b*_indirect_ = − .01, *SE*_boot_ = .03, 95% CI_boot_ [− .07, .05]), and psychological well-being (*b*_indirect_ = − .01, *SE*_boot_ = .02, 95% CI_boot_ [− .06, .04]).

Inconsistent with Hypothesis 4a, mediation analyses showed that self-rumination did not mediate the relationship between health motivation and psychological health.

If excluding vegetarians whose primary motivation is neither health nor morality, the results were consistent.

When the three disordered eating measures and self-rumination were entered in the model, the direct effect of health FCM was not significant on any measure of psychological health: depression (*b* = − .01, *SE*_boot_ = .05, *p* = .91), anxiety (*b* = − .06, *SE*_boot_ = .04, *p* = .16), stress (*b* = − .04, *SE*_boot_ = .04, *p* = .28), emotional well-being (*b* = .06, *SE*_boot_ = .07, *p* = .36), social well-being (*b* = .04, *SE*_boot_ = .07, *p* = .56), and psychological well-being (*b* = .11, *SE*_boot_ = .06, *p* = .10).

#### Subjective dietary adherence as a mediator

The results were summarized in Table 6.

**Table 6:** Mediation analysis testing the indirect effects of moral motivation on psychological health indicators via dietary adherence

Predictor	Outcome	Subjective adherence	Depression	Anxiety	Stress	EWB	SWB	PWB
		*b*	*b*	*b*	*b*	*b*	*b*	*b*
Constant		3.87*** (.50)	3.32*** (.43)	2.97*** (.34)	3.56*** (.37)	1.59** (.55)	1.15* (.65)	1.04 (.54)
Moral FCM		.12 (.07)	− .03 (.05)	.03 (.04)	− .02 (.04)	.04 (.07)	.05 (.08)	.06 (.06)
Subjective adherence			− .07 (.05)	− .10* (.04)	− .07 (.04)	.08 (.07)	.07 (.08)	.10 (.06)
Health FCM		− .001 (.06)	.07 (.05)	.03 (.04)	.04 (.04)	− .03 (.06)	.01 (.07)	.03 (.06)
Age		.01 (.05)	− .13** (.04)	− .16*** (.03)	− .15*** (.03)	.07 (.05)	.02 (.06)	.15** (.05)
Gender (0 = male)		.12 (.15)	.13 (.12)	.14 (.09)	.15 (.10)	.19 (.15)	− .24 (.17)	− .04 (.15)
Ethnicity (0 = non-White)		.31 (.18)	.21 (.14)	− .03 (.11)	.09 (.12)	− .19 (.18)	− .34 (.21)	− .09 (.18)
Urbanicity		− .01 (.05)	− .002 (.04)	.002 (.03)	− .02 (.03)	− .002 (.05)	− .01 (.05)	− .02 (.04)
Income		.03 (.05)	− .09* (.04)	− .08** (.03)	− .07* (.03)	.11* (.05)	.07 (.06)	.06 (.05)
Diet type (0 = non-strict vegetarian)		.79*** (.16)	.10 (.13)	− .08 (.10)	− .04 (.11)	− .10 (.17)	− .19 (.20)	− .29 (.17)
Physical health		.17* (.09)	− .21** (.07)	− .06 (.05)	− .16** (.06)	.44*** (.08)	.47*** (.10)	.45*** (.08)
Indirect effect via dietary adherence			− .01 (.01) [− .03, .005]	− .01 (.01) [− .03, .002]	− .01 (.01) [− .03, .003]	.01 (.01) [− .01, .03]	.01 (.01) [− .01, .04]	.01 (.01) [− .004, .04]

*EWB* Emotional well-being, *SWB* Social well-being, *PWB* Psychological well-being, *FCM* Food choice motive

*p < .05; **p < .01; ***p < .001

The indirect effects of moral FCM via dietary adherence on depression (*b*_indirect_ = − .01, *SE*_boot_ = .01, 95% CI_boot_ [− .03, .005]), anxiety (*b*_indirect_ = − .01, *SE*_boot_ = .01, 95% CI_boot_ [− .03, .002], and stress (*b*_indirect_ = − .01, *SE*_boot_ = .01, 95% CI_boot_ [− .03, .003]) were not significant. The indirect effects of moral FCM on measures of positive psychological health were not significant, either (emotional well-being: *b*_indirect_ = .01, *SE*_boot_ = .01, 95% CI_boot_ [− .01, .03]); social well-being: *b*_indirect_ = .01, *SE*_boot_ = .01, 95% CI_boot_ [− .01. .04]; psychological well-being: *b*_indirect_ = .01, *SE*_boot_ = .01, 95% CI_boot_ [− .004, .04]).

Inconsistent with Hypothesis 6a, the results showed that dietary adherence did not mediate the relationship between moral motivation and psychological health, possibly because moral motivation was not significantly correlated with dietary adherence after controlling for covariates.

Of note, diet type was significantly correlated with dietary adherence even after controlling for moral motivation and other covariates, such that consuming a strict vegetarian diet (vs. non-strict vegetarian diet) was associated with better dietary adherence, which in turn was associated with lower anxiety; the indirect effect of diet type on anxiety via dietary adherence was significant, *b*_indirect_ = − .08, *SE*_boot_ = .04, 95% CI_boot_ [− .16, − .01].

If excluding vegetarians whose primary motivation is neither health nor morality, the results were consistent.

#### Prosocial motivation and prosocial behavior as mediators

Autonomous prosocial motivation and prosocial behavior were entered as parallel mediators. The results were summarized in Table 7.

**Table 7:** Mediation analysis testing the indirect effects of moral motivation on psychological health indicators via autonomous prosocial motivation and prosocial behavior

Predictor	Outcome	Prosocial motivation	Prosocial behavior	Depression	Anxiety	Stress	EWB	SWB	PWB
		*b*	*b*	*b*	*b*	*b*	*b*	*b*	*b*
Constant		2.56*** (.51)	1.05** (.40)	2.97*** (.41)	2.36*** (.32)	3.06*** (.35)	1.69** (.52)	.85 (.57)	.97 (.50)
Moral FCM		.40*** (.07)	.14** (.05)	− .05 (.06)	− .02 (.04)	− .07 (.04)	.03 (.07)	− .01 (.08)	.01 (.07)
Prosocial motivation				.04 (.05)	.05 (.04)	.07 (.04)	.01 (.07)	.02 (.07)	.07 (.06)
Prosocial behavior				− .03 (.07)	.09 (.05)	.05 (.06)	.19* (.08)	.49*** (.09)	.25** (.08)
Health FCM		.07 (.06)	.06 (.05)	.07 (.05)	.02 (.04)	.03 (.04)	− .04 (.06)	− .02 (.07)	.01 (.06)
Age		.07 (.05)	− .06 (.04)	− .14*** (.04)	− .16*** (.03)	− .16*** (.03)	.09 (.05)	.05 (.06)	.16** (.05)
Gender (0 = male)		.11 (.16)	.26* (.12)	.12 (.12)	.10 (.09)	.12 (.10)	.15 (.15)	− .35* (.17)	− .10 (.14)
Ethnicity (0 = non-White)		.10 (.19)	− .02 (.15)	.18 (.14)	− .06 (.11)	.07 (.12)	− .16 (.18)	− .31 (.20)	− .06 (.17)
Urbanicity		− .05 (.05)	.01 (.04)	.001 (.04)	.01 (.03)	− .02 (.03)	− .005 (.05)	− .02 (.05)	− .02 (.04)
Income		.11* (.05)	.09* (.04)	− .09* (.04)	− .09** (.03)	− .08* (.03)	.10* (.05)	.03 (.05)	.03 (.05)
Diet type (0 = non-strict vegetarian)		.05 (.17)	− .27* (.13)	.04 (.13)	− .14 (.10)	− .09 (.11)	.02 (.16)	− .01 (.18)	− .14 (.16)
Physical health		− .05 (.09)	.12 (.07)	− .22** (.07)	− .09 (.05)	− .17** (.06)	.43*** (.08)	.43*** (.09)	.44*** (.08)
Indirect effect via moral motivation				.02 (.02) [− .03, .07]	.02 (.02) [− .01, .05]	.03 (.02) [− .01, .07]	.003 (.03) [− .05, .06]	.01 (.03) [− .05, .07]	.03 (.03) [− .02, .08]
Indirect effect via moral behavior				− .004 (.01) [− .03, .02]	.01 (.01) [− .003, .04]	.01 (.01) [− .01, .03]	.03 (.02) [.002, .06]	.07 (.03) [.02, .13]	.04 (.02) [.01, .07]

*EWB* Emotional well-being, *SWB* Social well-being, *PWB* Psychological well-being, *FCM* Food choice motive

*p < .05; **p < .01; ***p < .001

Unexpectedly, autonomous prosocial motivation did not mediate the relationship between moral motives and any measure of psychological health (depression: *b*_indirect_ = .02, *SE*_boot_ = .03, 95% CI_boot_ [− .03, .07]; anxiety: *b*_indirect_ = .02, *SE*_boot_ = .02, 95% CI_boot_ [− .01, .05]; stress: *b*_indirect_ = .03, *SE*_boot_ = .02, 95% CI_boot_ [− .01, .07]; emotional well-being: *b*_indirect_ = .003, *SE*_boot_ = .03, 95% CI_boot_ [− .05, .06]; social well-being: *b*_indirect_ = .01, *SE*_boot_ = .03, 95% CI_boot_ [− .05, .07]; psychological well-being: *b*_indirect_ = .03, *SE*_boot_ = .03, 95% CI_boot_ [− .02, .08]). For prosocial behavior, the indirect effects on depression (*b*_indirect_ = − .004, *SE*_boot_ = .01, 95% CI_boot_ [− .03, .02]), anxiety (*b*_indirect_ = .01, *SE*_boot_ = .01, 95% CI_boot_ [− .003, .04]), and stress (*b*_indirect_ = .01, *SE*_boot_ = .01, 95% CI_boot_ [− .01, .03]) were not significant. On the other hand, moral motivation had significant indirect effects via prosocial behavior on emotional well-being (*b*_indirect_ = .03, *SE*_boot_ = .02, 95% CI_boot_ [.002, .06]), social well-being (*b*_indirect_ = .07, *SE*_boot_ = .03, 95% CI_boot_ [.02, .13]), and psychological well-being (*b*_indirect_ = .04, *SE*_boot_ = .02, 95% CI_boot_ [.01, .07]).

Consistent with Hypothesis 7a, moral motivation was positively associated with prosocial *behavior*, which in turn was positively associated with emotional, social, and psychological well-being. Contrary to Hypothesis 7a, autonomous prosocial *motivation* was not a significant mediator of the relationship between moral motivation and psychological health.

If excluding vegetarians whose primary motivation is neither health nor morality, the results were consistent.

When dietary adherence, prosocial motivation, and prosocial behavior were included in the model, the direct effect of moral FCM was not significant on any measure of psychological health: depression (*b* = − .04, *SE*_boot_ = .06, *p* = .42), anxiety (*b* = − .01, *SE*_boot_ = .04, *p* = .81), stress (*b* = − .06, *SE*_boot_ = .05, *p* = .21), emotional well-being (*b* = .02, *SE*_boot_ = .07, *p* = .81), social well-being (*b* = − .02, *SE*_boot_ = .08, *p* = .78), and psychological well-being (*b* = − .003, *SE*_boot_ = .07, *p* = .97).

### Group comparisons

Mean differences between health vegetarians, moral vegetarians, and omnivores in the studied variables were examined by performing ANCOVA, with gender, age, ethnicity, household income, urbanicity, and physical health controlled for (see Table 8).

**Table 8:** One-way analysis of covariance in focal variables while controlling for age, gender, ethnicity, household income, urbanicity, and physical health

Measure	Moral vegetarians		Health vegetarians		Omnivores		*F*(2, 291)	*η*^2^
	*M*	SE	*M*	SE	*M*	SE		
Orthorexia	2.39_a_	.06	2.79_b_	.07	2.05_c_	.06	32.27***	.18
Dietary restraint	2.92_ab_	.09	3.15_a_	.11	2.74_b_	.10	3.91*	.03
Inflexible eating	3.00_a_	.09	3.44_b_	.11	2.62_c_	.10	15.60***	.10
Self-rumination	3.34_a_	.09	3.34_a_	.11	3.16_a_	.10	1.12	.01
Dietary adherence^a^	6.28_a_	.10	6.09_a_	.13			1.24	.01
Autonomous prosocial motivation	5.78_a_	.11	5.59_ab_	.14	5.34_b_	.12	3.38*	.02
Prosocial behavior	2.60_a_	.08	2.67_a_	.10	2.59_a_	.09	.18	.001
Depression	1.90_a_	.08	1.79_a_	.09	1.73_a_	.09	1.13	.01
Anxiety	1.61_a_	.06	1.60_a_	.07	1.43_a_	.07	2.19	.01
Stress	1.90_ab_	.07	1.94_a_	.08	1.67_b_	.07	4.10*	.03
Emotional well-being	4.30_a_	.10	4.23_a_	.12	4.29_a_	.11	.09	.001
Social well-being	3.35_a_	.11	3.21_a_	.14	3.29_a_	.13	.26	.002
Psychological well-being	4.07_a_	.10	4.09_a_	.12	4.10_a_	.11	.02	.0002

Means reported in the table are estimated marginal means. Means within the same row that do not share a subscript differ significantly in pairwise post-hoc comparisons with a Bonferroni correction

^a^When the outcome variable is dietary adherence, degrees of freedom were different: *F*(1, 197)

*p < .05; **p < .01; ***p < .001

As expected, there was a significant group difference in orthorexia, *F*(2, 291) = 32.27, *p* < .001. Pairwise comparisons with Bonferroni correction revealed that health vegetarians (*M* = 2.79, *SE* = .07) had higher levels of orthorexia than moral vegetarians (*M* = 2.39, *SE* = .06) and omnivores (*M* = 2.05, *SE* = .06); moral vegetarians (*M* = 2. 39, *SE* = .06) scored higher on orthorexia than omnivores (*M* = 2.05, *SE* = .06). Dietary restraint also significantly differed between groups, *F*(2, 291) = 3.91, *p* < .05. Pairwise comparisons suggested that health vegetarians (*M* = 3.15, *SE* = .11) had higher levels of dietary restraint than omnivores (*M* = 2.74, *SE* = .10); moral vegetarians (*M* = 2.92, *SE* = .09) did not significantly differ from health vegetarians (*M* = 3.15, *SE* = .11) and omnivores (*M* = 2.74, *SE* = .10). Results also revealed a significant group difference in inflexible eating, *F*(2, 291) = 15.60, *p* < .001. Pairwise comparisons showed that health vegetarians (*M* = 3.44, *SE* = .11) displayed higher levels of inflexible eating than moral vegetarians (*M* = 3.00, *SE* = .09) and omnivores (*M* = 2.62, *SE* = .10); moral vegetarians had higher levels of inflexible eating (*M* = 3.00, *SE* = .09) than omnivores (*M* = 2.62, *SE* = .10).

There was no significant group difference in self-rumination, *F*(2, 291) = 1.12, *p* = .33.

Unexpectedly, there was no significant difference in dietary adherence between health and moral vegetarians, *F*(1, 197) = 1.24, *p* = .27. The results were consistent if controlling for diet type.

A comparison of autonomous prosocial motivation suggested a significant group difference, *F*(2, 291) = 3.38, *p* < .05. Moral vegetarians (*M* = 5.78, *SE* = .11) showed significantly higher autonomous prosocial motivation than omnivores (*M* = 5.34, *SE* = .12); health vegetarians (*M* = 5.59, *SE* = .14) did not significantly differ from moral vegetarians or omnivores. On the other hand, there was no significant group difference in prosocial behavior, *F*(2, 291) =  .18, *p* = .83.

The results suggested no significant group difference in depression (*F*(2, 291) = 1.13, *p* = .33) and anxiety (*F*(2, 291) = 2.19, *p* = .11). However, levels of stress significantly differed among the three groups, *F*(2, 291) = 4.10, *p* < .05. Pairwise comparisons indicated lower stress levels in omnivores (*M* = 1.67, *SE* = .07) than in health vegetarians (*M* = 1.94, *SE* = .08); moral vegetarians (*M* = 1.90, *SE* = .07) did not significantly differ from health vegetarians (*M* = 1.94, *SE* = .08) and omnivores (*M* = 1.67, *SE* = .07) in stress. Of note, the difference between moral vegetarians and omnivores in stress was marginally significant, *p* = .05.

There was no significant group difference in terms of emotional well-being (*F*(2, 291) =  .09, *p* = .91), social well-being (*F*(2, 291) =  .26, *p* = .77), and psychological well-being (*F*(2, 291) =  .02, *p* = .98).

In summary, Hypotheses 1b and 3b were supported.

Consistent with Hypothesis 1b, health vegetarians had higher levels of orthorexia than moral vegetarians. Contrary to 2b, moral and health vegetarians did not significantly differ in dietary restraint. Consistent with Hypothesis 3b, health vegetarians displayed higher levels of inflexible eating than moral vegetarians. Contrary to Hypothesis 4b, moral and health vegetarians did not significantly differ in self-rumination. Contrary to Hypothesis 6b, moral and health vegetarians did not significantly differ in dietary adherence. Contrary to Hypothesis 7b, moral and health vegetarians did not significantly differ in autonomous prosocial motivation or prosocial behavior. Contrary to Hypothesis 8b, moral and health vegetarian did not significantly differ in psychological health.

## Discussion

### General discussion

Prior research studying the relationship between vegetarianism and psychological health generated inconsistent results and findings. One possible reason for this inconsistency could be that vegetarianism driven by health versus moral motivation is associated with differential psychological health, yet this possibility has not been systematically tested. To fill this gap, the current research examined whether health and moral motivation among vegetarians is associated with differential psychological and behavioral characteristics which may ultimately predict better or poorer psychological health.

It was hypothesized that health motivation would be associated with three markers of disordered eating: orthorexia, dietary restraint, and inflexible eating, which would in turn predict poorer psychological health. As expected, mediation analyses revealed that health motivation was positively correlated with dietary restraint and inflexible eating, which in turn predicted poorer psychological health; by contrast, orthorexia did not mediate the relationship between health motivation and any indicator of psychological health.

A positive relationship between health motivation and self-rumination was expected. Contrary to our hypothesis, health motivation was not significantly correlated with self-rumination after controlling for covariates. The null finding could be due to the way self-rumination was measured in the current study: self-rumination was measured in broad terms instead of being specific to rumination about physical health and bodily symptoms. Future research can test whether health motivation is associated with *health-related* rumination among vegetarians.

We also theorized that moral motivation among vegetarians would be positively correlated with dietary adherence and prosociality (i.e., autonomous prosocial motivation and prosocial behavior), which would in turn predict better psychological health. Contrary to our hypothesis, moral motivation did not significantly predict dietary adherence after controlling for covariates. Surprisingly, it was incidentally found that diet type was significantly correlated with dietary adherence after controlling for covariates, such that consuming a strict (vs. non-strict) vegetarian diet was associated with better dietary adherence, which in turn was associated with lower anxiety. These findings suggest that the type of diet a vegetarian adopts may play a more prominent role in their dietary adherence than their moral motivation, and this might account for the null association between moral motivation and dietary adherence after controlling for diet type (and other covariates). On the other hand, parallel mediation analysis showed that prosocial behavior (but not autonomous prosocial motivation) mediated that relationship between moral motivation and indicators of positive psychological health.

Although health and moral motivation was *indirectly* associated with differential psychological health, neither health motivation, nor moral motivation, had a significant total effect on any indicators of psychological health after controlling for covariates. The null findings on the direct relationships suggest that the current investigation on the role of mediating variables in the relationship between health/moral motivation and psychological health is theoretically and practically worthwhile. The null direct relationship findings also echo the mixed results concerning the link between vegetarianism and psychological health in the existing literature. The present research examined some of these mediating mechanisms, but there could be other psychological differences that are relevant for health and moral vegetarians. For example, one relevant psychological variable for moral vegetarians is empathy. Empathy, when not coupled with emotional regulation skills, can result in high levels of personal distress and unfavorable relational outcomes [74]. Therefore, we speculate that high levels of empathy experienced by moral vegetarians is a double-edged sword that can promote well-being by motivating prosocial behavior, and yet might be linked to greater interpersonal stress among those inadept at emotional regulation [27, 69].

Quite importantly, the null results on the direct relationship could be due to the fact that we did not differentiate between vegetarian and vegans in our analyses. As vegetarians and vegans may differ in their levels of endorsement of health or moral motivation and have different psychological experiences related to their dietary choice [61], the type of vegetarian diet (i.e., vegetarian vs. vegan) may confound the results. However, given the small sample pool of vegan participants (*N* = 81), we could not conduct analyses to reliably detect differences between the vegetarian and vegan groups.

Another possible explanation for the null results is that health motivation and moral motivation is associated with different personality profiles among vegetarians [36], which may have important implications on their psychological health outcomes. Future research can control for personality traits when examining the association between motivation and psychological health among vegetarians.

### Theoretical contributions

Despite null findings regarding the total effects of health/moral motivation on psychological health, the current research yielded some interesting findings that are worthy of attention. First, moral vegetarians had marginally significantly higher levels of stress than omnivores (*p* = .05); health vegetarians had significantly higher levels of stress than omnivores. On the other hand, moral vegetarians, health vegetarians, and omnivores did not significantly differ on other measures of psychological health. Second, mediation analyses shed light upon the mechanisms that could affect psychological distress and positive well-being among vegetarians. Health motivation was positively associated with dietary restraint and inflexible eating; while dietary restraint was in turn associated with higher levels of anxiety and stress, inflexible eating was in turn associated with lower levels of emotional well-being. For vegetarians with higher health motivation, their increased dietary restraint may act as a risk factor for increased psychological distress, and their increased inflexible eating may reduce feelings of positive well-being. On the other hand, moral motivation was positively correlated with prosocial behavior, which in turn predicted higher levels of emotional, social, and psychological well-being. For vegetarians with higher moral motivation, their greater engagement in prosocial behavior may promote positive well-being. Lastly, although moral motivation was not associated with better dietary adherence after controlling for covariates, we incidentally found that a strict (vs. non-strict) vegetarian diet was associated with better dietary adherence, which in turn was associated with less anxiety.

These findings can have implications for developing effective interventions for promoting psychological health among vegetarians. Health practitioners can be more mindful of the specific psychological and behavioral correlates of different dietary motives. For example, dietary restraint and inflexible eating as risk factors for compromised psychological health are uniquely associated with health (but not moral) motivation among vegetarians. With this information, interventions aiming to alleviate emotional distress among vegetarians with strong health-related motives should target dietary restraint and inflexible focus on dietary rules. On the other hand, to promote psychological health of vegetarians with strong moral motives, health practitioners may encourage them to translate their autonomous prosocial motivation into actual prosocial behavior, as the current research suggests that prosocial behavior (but not autonomous prosocial motivation) predicted better well-being among vegetarians. Finally, given that consuming a strict (vs. non-strict) vegetarian diet was indirectly associated with lower anxiety via increased dietary adherence, health practitioners may encourage the adoption of a strict vegetarian diet among those vegetarians who eat a non-strict vegetarian diet and feel highly anxious about not being able to adhere to their chosen diet.

### Study limitations

Although the present research yielded some important findings, it has several limitations. First, despite revealing psychological/behavioral correlates of health and moral motivation which can have implications on psychological health among vegetarians, the study’s cross-sectional design makes it difficult to draw conclusions about the temporal and causal relations between the development of health-motivated/morality-motivated vegetarianism and people’s psychological health states. In the present research, health vegetarians had significantly higher levels of stress than omnivores; moral vegetarians had marginally significantly higher levels of stress than omnivores. This finding does not necessarily indicate that morality-motivated vegetarianism and health-motivated vegetarianism will *lead to* poorer psychological health. A study based on retrospective reports [51] found that the onset of mental disorders tended to precede the adoption of vegetarianism. Therefore, it is possible that people with poor psychological health try to enhance their well-being by engaging in activities and pursuing goals which they consider as meaningful (i.e., pursing a moral life or pursuing better physical health by eating a vegetarian diet). Whether pursuing a moral- or health-related goal may have implications on the effectiveness of this coping strategy. Future research can employ a longitudinal design and recruit prospective vegetarians in order to trace the trajectory of psychological health of individuals who eat a non-vegetarian diet at the beginning but later adopt a vegetarian diet for moral motives versus health motives.

Second, the measurement of prosocial behavior is broad and not diet-relevant. It is possible that moral motivation among vegetarians is more strongly associated with diet-related prosocial behavior (vs. general prosocial behavior). Future research may examine the relationship between moral motivation and diet-related prosocial behavior (e.g., reduce food waste, consume locally grown food, buy food of brands with a prosocial image), and whether engaging in diet-related prosocial behavior is associated with better psychological health.

Third, in the present research participants were asked to select only one predominant motivation for vegetarianism and were classified into health vegetarians and moral vegetarians accordingly. This aimed to identify the most important motivation for participants to adopt a vegetarian diet. However, this classification does not take into account those vegetarians equally weighing health and moral factors, which may apply to some individuals in the vegetarian group [61]. Future research can provide the option for vegetarian participants to select more than one predominant motivation for vegetarianism, and compare health vegetarians, moral vegetarians, and vegetarians who are equally motivated by health and moral concerns.

Fourth, considering that moral vegetarians tend to value their diet-related identity to a greater extent than health vegetarians [58], it could be argued that moral vegetarians might be more motivated to respond in a way that presents a psychologically healthy view of vegetarians. However, this likelihood is diminished for two reasons. Firstly, participants were informed that the research was investigating their lifestyle (i.e., dietary preferences and habits, and helping behavior), experience as a vegetarian, and physical and psychological health. This description may obscure the purpose of this study. Additionally, the results showed that moral vegetarians reported marginally significantly higher levels of stress compared to omnivores. This finding suggested that moral vegetarians may not intentionally present their identity in a positive light.

Another limitation is that the current sample is small and not gender-balanced. Although the current research did not examine gender differences, it is likely that the relationship between health motivation and psychological health may differ across gender. Health motivation could be more strongly correlated with dietary restraint and inflexible eating among female (vs. male) vegetarians, given that females might be more susceptible to disordered eating. Future study may examine the relationship between health/moral motivation and psychological health across gender and in a larger sample.

## Conclusion

While health and moral motivation could be associated with disparate psychological and behavioral tendencies, the association between dietary motivation and psychological health among vegetarians is rarely studied. The present research demonstrated that health and moral motivation was associated with differential psychological and behavioral characteristics, which in turn predicted differential psychological health indicators among vegetarians. Specifically, health motivation was positively correlated with disordered eating tendencies, indirectly linking it to poorer psychological health; moral motivation was positively with prosocial behavior, which in turn predicted better psychological health. These findings may provide important insights for the development of interventions that aim to promote the psychological health of vegetarians.

## Data Availability

The data and code for this research can be accessed at: https://data.mendeley.com/datasets/n4kd8pyx2g/2.
